# Supplementary material for: Typologies of childhood maltreatment and associations with internalizing symptoms among university students in Singapore: A latent class analysis
Source: PLoS One. 2025 Apr 11;20(4):e0320851. doi: 10.1371/journal.pone.0320851 (PMC11990771; doi:10.1371/journal.pone.0320851)
Supplement: Attachment — Submitted filename: Response to Reviewers_0206.docx [file pone.0320851.s003.docx]

Dear Editor,

Thank you for this opportunity to revise and resubmit the manuscript entitled “Typologies of Childhood Maltreatment and Associations with Internalizing Symptoms among Young Adults in Singapore: A Latent Class Analysis” (PONE-D-24-24662) to *PLOS ONE*. We appreciate the reviewer’s comments which have guided us to improve the quality of the paper. In this letter, the authors are providing point-by-point responses to all the reviewer’s comments.

This revised manuscript contains original material and has not been submitted for review elsewhere. If I can provide any information that would be of use to editorial staff, please do not hesitate to contact me.

Thank you in advance for your time and effort in the review of this manuscript, and I appreciate your consideration. I look forward to hearing from you soon.

**Response to the editorial comments**

Journal requirements:

1. When submitting your revision, we need you to address these additional requirements.

Please ensure that your manuscript meets PLOS ONE's style requirements, including those for file naming. The PLOS ONE style templates can be found at

https://journals.plos.org/plosone/s/file?id=wjVg/PLOSOne_formatting_sample_main_body.pdf and

https://journals.plos.org/plosone/s/file?id=ba62/PLOSOne_formatting_sample_title_authors_affiliations.pdf

[Author Response]: We carefully followed PLOS ONE’s style. Thank you!

2. Thank you for stating the following financial disclosure:

“This work was supported by Start-Up Grant from the National University of Singapore [Grant No. R134-000-098-133].”

Please state what role the funders took in the study.  If the funders had no role, please state: "The funders had no role in study design, data collection and analysis, decision to publish, or preparation of the manuscript."

If this statement is not correct you must amend it as needed.

Please include this amended Role of Funder statement in your cover letter; we will change the online submission form on your behalf.

[Author Response]: We have amended the Role of Funder statement in our cover letter by including the statement, “The funders had no role in study design, data collection and analysis, decision to publish, or preparation of the manuscript."

3. In this instance it seems there may be acceptable restrictions in place that prevent the public sharing of your minimal data. However, in line with our goal of ensuring long-term data availability to all interested researchers, PLOS’ Data Policy states that authors cannot be the sole named individuals responsible for ensuring data access (http://journals.plos.org/plosone/s/data-availability#loc-acceptable-data-sharing-methods).

Data requests to a non-author institutional point of contact, such as a data access or ethics committee, helps guarantee long term stability and availability of data. Providing interested researchers with a durable point of contact ensures data will be accessible even if an author changes email addresses, institutions, or becomes unavailable to answer requests.

Before we proceed with your manuscript, please also provide non-author contact information (phone/email/hyperlink) for a data access committee, ethics committee, or other institutional body to which data requests may be sent. If no institutional body is available to respond to requests for your minimal data, please consider if there any institutional representatives who did not collaborate in the study, and are not listed as authors on the manuscript, who would be able to hold the data and respond to external requests for data access? If so, please provide their contact information (i.e., email address). Please also provide details on how you will ensure persistent or long-term data storage and availability.

[Author Response]: There is no non-author institutional point of contact to access the data. The authors are sharing the original data as a supplementary material and the availability of data and materials statement are revised as follows:

“Availability of data and materials

The data that support the findings of this study are available as a supplementary file. However, we encourage those researchers who plan to use this data for the analysis to clearly cite the source.”

4. Please amend either the title on the online submission form (via Edit Submission) or the title in the manuscript so that they are identical.

[Author Response]: Amended the title identically.

5. Your ethics statement should only appear in the Methods section of your manuscript. If your ethics statement is written in any section besides the Methods, please move it to the Methods section and delete it from any other section. Please ensure that your ethics statement is included in your manuscript, as the ethics statement entered into the online submission form will not be published alongside your manuscript.

[Author Response]: The ethics statement is already included in the Methods section. However, the authors added the IRB number and how the consent was obtained from the individuals (pp.7-8). The ethics statement which was initially located at the bottom of the manuscript is now deleted and it is only being discussed in the Methods section.

“To test the research hypotheses, the present study employed a cross-sectional survey design and recruited university students from a major university in Singapore using purposive sampling. Participants were eligible for the study if they were full-time students, between 21 and 30 years old, pursuing an undergraduate or graduate degree, using information and communications technology in everyday life, and English-speaking. Participants received an email invitation to participate in an online survey examining childhood and current trauma experiences, as well as mental health conditions. Data were collected via the university’s eSurvey platform. The survey participation was anonymous and voluntary. Given the online survey, participants’ informed consent was obtained in the following manner. Once the participants accessed the survey link, it led them first to a consent form, which outlines important details about the study, such as the purpose of the study, confidentiality, target population, the expected duration, and the possible benefits and risks associated with participation. If, after reading the consent form in full, respondents decided to participate in the survey, they clicked the “I Agree” button, indicating their informed consent and thereby leading them to the first page of the online survey (A waiver of the documentation of informed consent – no documented consent). That is, no documented consent form was collected. Instead, the participants consented to participate in the study by completing the enclosed online survey. The recruitment period for this study was between 1 August 2019 and 31 December 2019. After data collection, any personally identifiable information was deleted, and non-identifiable data were securely stored in an encrypted drive. Prior to initiating the online survey, study procedures were reviewed and approved by a university Institutional Review Board in accordance with the Declaration of Helsinki (NUS-IRB reference number: LS-18-201).”

6. Please include a separate caption for each figure in your manuscript.

[Author Response]: There is only one figure used in this manuscript and the authors added the caption for this figure as follows:

“<<Insert Figure-1: Item-Response Probabilities for Seven Childhood Traumatic Experiences for the Four Latent Classes, about here>>”

Comments from PLOS Editorial Office: We note that one or more reviewers has recommended that you cite specific previously published works. As always, we recommend that you please review and evaluate the requested works to determine whether they are relevant and should be cited. It is not a requirement to cite these works. We appreciate your attention to this request.

[Author Response]: We make sure to review the works before citing them in our manuscript. Thank you.

**Response to the reviewer’s comments**

**Reviewer 1:**

1. There are few references in recent five years, so it is recommended to check and update the literature to ensure the comprehensiveness and timeliness of the literature review.

[Author Response]: Thanks for your advice. We have updated with several new references as per your comments. Here are some of the recent references we added to the Introduction:

Hecker T, Boettcher VS, Landolt MA, Hermenau K. Child neglect and its relation to emotional and behavioral problems: A cross-sectional study of primary school-aged children in Tanzania. Development and Psychopathology. 2018 Mar;31(1):325-339.

Ainamani HE, Weierstall-Pust R, Bahati R, Otwine A, Tumwesigire S, Rukundo G. Post-traumatic stress disorder, depression and the associated factors among children and adolescents with a history of maltreatment in Uganda. European Journal of Psychotraumatology. 2022 Jan;13(1):1-11.

Henry LM, Gracey K, Shaffer A, Ebert J, Kuhn T, Watson KH, et al. Comparison of three models of adverse childhood experiences: Associations with child and adolescent internalizing and externalizing symptoms. Journal of Abnormal Psychology. 2021 Jan;130(1):9-25.

Bevilacqua L, Kelly Y, Heilmann A, Priest N, Lacey RE. Adverse childhood experiences and trajectories of internalizing, externalizing, and prosocial behaviors from childhood to adolescence. Child Abuse Neglect. 2021 Feb;112:104890.

Leban L. The effects of adverse childhood experiences and gender on developmental trajectories of internalizing and externalizing outcomes. Crime & Delinquency. 2021 Jan;67(5):1-24.

Grigsby TJ, Rogers CJ, Albers LD, Benjamin SM, Lust K, Eisenberg ME, et al. Adverse childhood experiences and health indicators in a young adult, college student sample: Differences by gender. International Journal of Behavioral Medicine. 2020 Jul;27:660-667.

2. In the introduction, the author mentions the use of Asian samples as one of the uniqueness and value of the study, but in the paper, there is less explanation of the comparison and possible mechanism of the similarities and differences between Eastern and Western subjects.

[Author Response]: Thank you for the comment. The authors would like to clarify that the inclusion of Asian samples is not the unique feature of this study. Since the current statement is misleading, we made the following adjustments (pp.3-4): “For example, only a few studies determined the relationship between the typologies of adverse childhood experiences (ACEs) and internalizing problems/disorders (see Lew & Xian^4^ for U.S. children).”

3. With more than twice as many women as men in the study sample and more than 10 times as many Chinese as non-Chinese, there is an imbalance in the grouping that may have had some influence on the results.

[Author Response]: Thank you for the feedback. The authors are fully aware that the study sample did not achieve gender and ethnicity proportionality, which may have impacted the results and the interpretation of the findings. As a result, the authors included this as a limitation of the study (p.22):

“Due to limitations in obtaining samples, there are twice as many females as males in the study sample and more than 10 times as many Chinese as non-Chinese. There is an imbalance in the groupings which could impact the results and thus, the results cannot accurately represent the Singaporean sample. Future studies should develop a sampling strategy that could achieve proportionality in both gender and ethnicity.”

4. There are cases where writing is not rigorous, for example, page 4, latent class analysis (LCA) mixed with LCA; Page 8, The average monthly household income was 4.73 (SD=1.62; range 1-7). Lack of units of average monthly income.

[Author Response]: Thanks for your comments. We have revised the cases to have their full word and acronym rigorously (e.g., latent class analysis and LCA, adverse childhood experiences and ACEs). In addition, we have updated the average monthly household income.

**Reviewer 2:**

Introduction
1. The intro discusses that CTEs are related to increased eating disorder rates, but I’m wondering if there will be an exploration of which EDs, given there is a diversity of them (e.g., anorexia to binge eating). This is also relevant for the methods section when the EDQ is discussed. I’m wondering if that has been used to assess all Eds, or those more related to body image and restriction, like anorexia or bulimia. However, EDs like Avoidant Restriction Food Intake Disorder (ARFID) and binge eating may not be as relevant. All this to say it may be important to classify or operationalize EDs more specifically throughout the manuscript.
[Author Response]: Thank you for your insightful comment. The current study did not use a clinical scale to measure EDs, as the participants are not from a clinical population. ED was assessed by the 28-item EDE-Q (version 6.0) that consists of 4 subscales: dietary restraint, eating concern, shape concern, and weight concern), and we used a global score that is the average of the 4 subscale scores to measure university students’ eating disorder symptoms. The measurement for eating disorders is a questionnaire that has high internal consistency and test-retest reliability, and it is used in a few other published studies that are reflected in the Introduction and Methods (under *Measures*) sections. It is also considered the preferred instrument for assessing and diagnosing eating disorders, as outlined in the fourth edition of the Diagnostic and Statistical Manual of Mental Disorders (DSM-IV).

In this study, the authors did not explore the relationship between each subscale of the questionnaire and CM, and as such, will not be discussing this in the Introduction and Discussion sections. However, we note that different types of EDs may have different relationships with CM, and this would be an interesting area of exploration for future studies. We have included this under the Limitations section (see pp. 21-22).

“In particular, this study did not explore the association of each subscale of eating disorder measure (EDE-Q 6.0) with CM. Therefore, future research would benefit to explore how CM is related to different types of eating disorders.”

Methods
2. Women/men would be more appropriate if gender is being explored, whereas male/female would fit best if sex is the demographic variable of interest

[Author Response]: Thank you for the advice. As gender was being explored in this study, we have amended to women/men in the manuscript accordingly.

Results
3. It states “Insert Table 1, about here” on p. 12 of the manuscript. I’m unsure if this is an error or a formatting reference to Tables in the supplemental documents. Similar for tables and figures later on
[Author Response]: Thank you for the comment. Now, all the Tables are located within the text instead of being placed at the bottom of the manuscript.

4. The presentation of covariates relationship to the outcome variables would make sense to present prior to the linear regression results that contain the demographic variables as covariates.
[Author Response]: Thank you for the comment. We first presented the relationship between covariates and the outcome variables and then presented the main linear regression results after controlling for the demographic variables.

5. Is it assumed that the 4 classes provided by the LCA are mutually exclusive? It can be that participants can fall into the categories of high/multiple CTEs and also abuse/victimization, for example. How does that influence data classification and interpretation?
[Author Response]: Thanks for your comment. In the current study, LCA assigned individual participants to the most probable latent class based on their response patterns. It means that the identified classes are mutually exclusive within the model. But this does not imply that individual participants are not able to exhibit characteristics of multiple classes. Instead, LCA categorizes them according to predominant response patterns they display.

For individual participants who might fit both “high/multiple CM” (revised “CTEs” to “CM”) and “moderate to high abuse/victimization” categories, the model assigns them to the best-fitting class as per probabilities. If there is conceptual overlap between these categories, it suggests that the identified classes may not be entirely different. This could indicate the need for alternative model specifications, including additional classes or higher-order classifications. Addressing these complexities in classification is crucial, as it can influence data interpretation and ensure that class definitions align with both theoretical frameworks and empirical evidence.

6. Is there a way to classify, or provide more information people who may fall in more than one typology as described by the LCA?
[Author Response]: Thank you for the insightful comment. We revised the typologies of the four latent classes and provided more information in the manuscript.

Discussion
7. It is a bit confusing to refer to the four latent classes as different typologies of CTE, given that two refer to frequency (high vs low) and refer to type of trauma experienced (neglect or abuse). I’m wondering if there is a different way to classify those, or if more explanation is warranted as to how those are characterized into typologies of CTE. Typologies of CTE may render readers to think more about CTQ subscales (neglect, abuse, physical, sexual, etc). It might make more sense to discuss characteristics of CTEs.
[Author Response]: Thank you for your feedback. As per your suggestions, the authors revised the typologies of the four latent classes: Class 1 = low CM; Class 2 = high/multiple CM; Class 3 = moderate to high abuse/victimization; Class 4 = moderate to high neglect. As Review 3 recommended to replace “CTE” to “Childhood maltreatment (CM)”, we also amended accordingly in the manuscript.

8. As authors discuss, associations are not a direct path. Thus, implications about sequential relationship between the variables may not be fully appropriate given the method of analysis and what is missing to attribute causality or even directionality. For example, it could be that individuals with existing mental health challenges elicit greater neglect or abuse from caregivers due to the stress of caring for them, or it could also be the other relationship. That nuanced, bidirectional relationship should be explored given the method of analysis rather than assuming the direction can be exacted with associations alone.
[Author Response]: Thank you for the feedback. The authors agree with the reviewer that discussing the LCA results in the context of a sequential relationship may not be appropriate. As such, we toned down our language in the discussion section to not make a direct assumption about the directionality (pp.19-20):

“Although the current study did not aim to identify the specific path from the history of CM to later mental health outcomes, a significant association between CM and mental health symptoms and outcomes further implies that childhood trauma, negative mental health symptoms, and psychiatric outcomes during adulthood can be explored in multiple directions with a set of mediator(s) or moderator(s). For example, CM was associated with poorer mental health functioning through negative posttraumatic cognitions among young adults.^55^ There were also instances where physical maltreatment and binge eating was mediated by psychological distress (e.g., depression, anxiety),^56^ and the relationship between emotional neglect or emotional abuse and suicide risk was mediated by depression.^57^ As such, more studies should be conducted to test the various forms of directionality among childhood maltreatment, mental health, and the associated internalizing outcomes in order to develop the most effective prevention and intervention programs that not only reduce traumatic experiences (e.g., abuse, neglect, victimization) for children, but also, boost the psychological well-being of young adult victims of CM.”

9. The discussion of eating disorders can be made more nuanced and details given the diverse presentation of EDs (see my earlier comment in introduction)
[Author Response]: Thank you for the feedback. As explained in the earlier response to your comment in Introduction, the current study did not use a clinical scale to measure EDs. ED was assessed by the 28-item EDE-Q (version 6.0) that consists of 4 subscales: dietary restraint, eating concern, shape concern, and weight concern), and we used a global score that is the average of the 4 subscale scores to measure university students’ eating disorder symptoms. The measurement for eating disorders is a questionnaire that has high internal consistency and test-retest reliability, and it is used in a few other published studies that are reflected in the Introduction and Methods (under *Measures*) sections. It is also considered the preferred instrument for assessing and diagnosing eating disorders, as outlined in the fourth edition of the Diagnostic and Statistical Manual of Mental Disorders (DSM-IV). Thus, we provided more nuanced and detailed information in the Introduction, Measurement, and Limitations section.

10. The discussion of mediators are helpful, but again limited given the study design.
[Author Response]: Thank you for the comment. Similar to our response in #8, the authors updated the paragraph to avoid getting into a deeper discussion about the mediators as that is not the main focus of this study (see pp.19-20):

“Although the current study did not aim to identify the specific path from the history of CM to later mental health outcomes, a significant association between CM and mental health symptoms and outcomes further implies that childhood trauma, negative mental health symptoms, and psychiatric outcomes during adulthood can be explored in multiple directions with a set of mediator(s) or moderator(s). For example, CM was associated with poorer mental health functioning through negative posttraumatic cognitions among young adults.^55^ There were also instances where physical maltreatment and binge eating was mediated by psychological distress (e.g., depression, anxiety),^56^ and the relationship between emotional neglect or emotional abuse and suicide risk was mediated by depression.^57^ As such, more studies should be conducted to test the various forms of directionality among childhood maltreatment, mental health, and the associated internalizing outcomes in order to develop the most effective prevention and intervention programs that not only reduce traumatic experiences (e.g., abuse, neglect, victimization) for children, but also, boost the psychological well-being of young adult victims of CM.”

11. The discussion briefly mentions how these results differ from western studies. This is important, but also requires more explanation as to why that is, or how it’s believed that these may be different.
[Author Response]: Thank you for the comment. This study did not have the intention to compare the current findings with those conducted in the Western countries. As there were a couple of misleading statements in the manuscript that might have confused the readers, the authors revised them throughout the manuscript.

12. I’m unsure that physical, material deprivation is not related to negative outcomes at all. I would specify that this is related to PTSD only as negative findings, but not with depression and anxiety. I think more information regarding why authors can understand the link between neglect and depression/anxiety would be helpful.

[Author Response]: Thank you for the feedback. The authors updated the paragraph so that it clarifies the significant association between physical and emotional neglect and depression and anxiety, but not with PTSD symptoms (pp.20-21):

“Meanwhile, physical and emotional neglect class was associated with the symptoms of depression, anxiety, eating disorder and suicidal ideation but not significantly associated with PTSD symptoms. This aligns with the results of the meta-analysis where pooled odds ratios for the association between neglect and depressive disorders, and neglect and anxiety disorders were significant, whereas its association with PTSD was not statistically significant.^58^ One possible speculation is that neglect is different from other forms of CM as it is closely tied to poverty. Since neglect usually involves the status when the caregivers are unable to meet the child’s basic needs, it may not always result in the child experiencing trauma. In the similar vein, Dorahy and colleagues^59^ postulate that physical neglect being the material deprivation and not necessarily emotional deprivation would have relatively less impact on children trauma-wise . Nevertheless, more evidence is required to clearly explain why physical and emotional neglect group may be related to depression and anxiety, but not with PTSD symptoms.”

**Reviewer 3:**

1. Title: The authors are advised to replace childhood traumatic experiences with Childhood maltreatment or Adverse childhood events/experiences.
[Author Response]: Thanks for your comment. We have replaced ‘childhood traumatic experiences’ to ‘childhood maltreatment’ in both title and text accordingly in accordance with your advice. As this study only focused on direct childhood maltreatment experiences (not indirect experiences such as dysfunctional household issues), the term ‘childhood maltreatment’ is more appropriate than ‘adverse childhood experiences’.

2. Abstract: I miss to see the measurement/assessment tools used in the study.
[Author Response]: The measurement/assessment tools this study used have been added in Abstract.

Review of Literature
3. The literature review provides a broad overview of the global and regional significance of identifying typologies of childhood traumatic experiences (CTEs) and their associations with CTEs with five internalizing symptoms in 1,042 university students in Singapore. It contextualizes the issue within the broader public health crisis and highlights the specific vulnerability of survivors of CTEs.
Although the review covers many relevant studies, it could include more recent works in the field of childhood maltreatment and trauma (eg. https://doi.org/10.1186/s13034-021-00373-7, https://doi.org/10.1080/20008198.2021.2007730)
[Author Response]: Thanks for your advice. We have updated with several new references, including two articles you suggested. Here are some of the recent references we added to the Introduction and Discussion:

Hecker T, Boettcher VS, Landolt MA, Hermenau K. Child neglect and its relation to emotional and behavioral problems: A cross-sectional study of primary school-aged children in Tanzania. Development and Psychopathology. 2018 Mar;31(1):325-339.

Ainamani HE, Weierstall-Pust R, Bahati R, Otwine A, Tumwesigire S, Rukundo G. Post-traumatic stress disorder, depression and the associated factors among children and adolescents with a history of maltreatment in Uganda. European Journal of Psychotraumatology. 2022 Jan;13(1):1-11.

Henry LM, Gracey K, Shaffer A, Ebert J, Kuhn T, Watson KH, et al. Comparison of three models of adverse childhood experiences: Associations with child and adolescent internalizing and externalizing symptoms. Journal of Abnormal Psychology. 2021 Jan;130(1):9-25.

Bevilacqua L, Kelly Y, Heilmann A, Priest N, Lacey RE. Adverse childhood experiences and trajectories of internalizing, externalizing, and prosocial behaviors from childhood to adolescence. Child Abuse Neglect. 2021 Feb;112:104890.

Leban L. The effects of adverse childhood experiences and gender on developmental trajectories of internalizing and externalizing outcomes. Crime & Delinquency. 2021 Jan;67(5):1-24.

Grigsby TJ, Rogers CJ, Albers LD, Benjamin SM, Lust K, Eisenberg ME, et al. Adverse childhood experiences and health indicators in a young adult, college student sample: Differences by gender. International Journal of Behavioral Medicine. 2020 Jul;27:660-667.

Ainamani HE, Rukundo GZ, Nduhukire T, Ndyareba E, Hecker T. Child maltreatment, cognitive functions and the mediating role of mental health problems among maltreated children and adolescents in Uganda. Child and Adolescent Psychiatry and Mental Health. 2021 Apr; 15:22.

Nweze T, Ezenwa M, Ajaelu C, Okoye C. Childhood mental health difficulties mediate the long-term association between early-life adversity at age 3 and poorer cognitive functioning at ages 11 and 14. Journal of Child Psychology and Psychiatry. 2023 Jun;64(6):952-965.

Identification of the gap
4. The manuscript correctly identifies the gap in the literature regarding childhood advance experiences. The focus on this specific population and the attempt to explore “Childhood Traumatic experiences” and their association with both externalized and internalized symptoms using latent classic analysis is commendable. The manuscript should however, clearly articulate why filling this gap is crucial for both academic knowledge and practical interventions.
[Author Response]: Thank you for your insightful comment. The authors made the following changes in the Introduction to clearly articulate the gap and benefits of this study in both research- and practice-levels (p.4):

“Assessing typologies of CM and its associations with internalizing symptoms has become an incumbent task for researchers where the knowledge would guide the direction of future studies, such as exploring preventive measures and effective intervention for high-risk groups. For professionals like clinicians, teachers, and caregivers of individuals who have experienced CM, understanding the typologies can guide them in directing resources toward those who need the most support, while also targeting the specific internalizing symptoms they are most likely to develop. Using a latent class analysis (LCA), the primary aims of this study are to identify the main different patterns of traumatic events that occur in childhood and how each type of CM is associated with internal symptoms among young adults, with the hope that the findings can promote efficient services for children and youth who have suffered from CM.”

Methods

5. The manuscript misses structural methodological practices (e.g., data entry and security, ethical considerations, design). The authors could consider including some of these subheadings.
[Author Response]: Thank you for your comments. The author added the research design with the purposive sampling, data entry and security, and ethical considerations in the section of Participants and Data Procedures even though we did not include subheadings.

6. The study uses well-established and standardized instruments like the “Center for Epidemiological Studies Depression Scale” Beck Anxiety Inventory, which adds to the methodological rigor. In relation to each measure, the authors should discuss reliability in this specific context.

[Author Response]: Thank you for the feedback. Like the reviewer mentioned, this study tried to use the standardized instruments to measure the constructs accurately. To demonstrate the methodological rigor, the authors did include the internal consistency. (reliability, Cronbach’s alpha) at the end of each measurement’s description.

7. It is shown that PTSD was assessed by a checklist. The authors could mention a specific name for this tool. What do the authors mean by PCL in the same paragraph?
Childhood traumatic experiences could be replaced by either maltreatment or Advance Childhood experiences. Maltreatment or ACES might not necessarily be traumatic.
Since the authors have opted to have a sub heading on the control variables, it would be beneficial `to have subheading on both Predictor and outcome variables as well.
[Author Response]: The PTSD Checklist (PCL) is the name of a validated self-report rating scale developed by Weathers et al. (1993). The PCL is the acronym for the PTSD Checklist. To avoid the confusion of it, the authors revised the PCL measurement part in the Measurement section.

Based on your comment, we replaced childhood traumatic experiences to childhood maltreatment. Finally, we added subheadings on the independent variable for childhood maltreatment and outcome variables for internalizing symptoms.

8. Results: The type of the regression model utilized in the analysis should be clearly stated in the analysis plan and table headings.
I appreciate the fact that the current table 1 presents the mean scores for the main study variables. However, the manuscript misses information on the social demographic characteristics.
[Author Response]: Thank you for the feedback. Based on the reviewer’s comments, the authors add the type of the regression model and stated more details in the analysis plan and table headings (please see the Statistical Analyses Section and Tables 1 and 4). We also added the information on the social demographic characteristics in Table 1 and the Results section, “Descriptive Statistics and Comparisons by Gender and Ethnicity”.

9. Presenting results on social demographic characteristics and intercorrelations between the continuous variables would benefit the manuscript.
[Author Response]: As responded in the above comment 8, the authors presented results on social demographic characteristics and intercorrelations between outcome variables in the Descriptive Statistics and Comparisons by Gender and Ethnicity section.

Discussion

The discussion is well written

Overall Assessment
10. This manuscript addresses an important subject of advance childhood experiences among the university students in Singapore. The study’s use of established standardized tools and appropriate statistical analyses adds rigor to the findings.

[Author Response]: Thank you for your overall comment.
